# Precision in Action: The Role of Clustered Regularly Interspaced Short Palindromic Repeats/Cas in Gene Therapies

**DOI:** 10.3390/vaccines12060636

**Published:** 2024-06-07

**Authors:** Amrutha Banda, Olivia Impomeni, Aparana Singh, Abdul Rasheed Baloch, Wenhui Hu, Dabbu Kumar Jaijyan

**Affiliations:** 1 Department of Biology, The College of New Jersey, Ewing Township, NJ 08618, USA; 2 Department of Chemistry, National Institute of Technology Agartala, Agartala 799046, India; aparanasingh02@gmail.com; 3 Department of Anatomy and Neurobiology, School of Medicine, Virginia Commonwealth University, Richmond, VA 23284, USA; abdulrasheed.khanzaibaloch@vcuhealth.org

**Keywords:** CRISPR/Cas, genetic disease, cancer, infection, genome editing, gene therapy, gene delivery, viral vectors

## Abstract

Clustered Regularly Interspaced Short Palindromic Repeat (CRISPR)-associated enzyme-CAS holds great promise for treating many uncured human diseases and illnesses by precisely correcting harmful point mutations and disrupting disease-causing genes. The recent Food and Drug Association (FDA) approval of the first CRISPR-based gene therapy for sickle cell anemia marks the beginning of a new era in gene editing. However, delivering CRISPR specifically into diseased cells in vivo is a significant challenge and an area of intense research. The identification of new CRISPR/Cas variants, particularly ultra-compact CAS systems with robust gene editing activities, paves the way for the low-capacity delivery vectors to be used in gene therapies. CRISPR/Cas technology has evolved beyond editing DNA to cover a wide spectrum of functionalities, including RNA targeting, disease diagnosis, transcriptional/epigenetic regulation, chromatin imaging, high-throughput screening, and new disease modeling. CRISPR/Cas can be used to engineer B-cells to produce potent antibodies for more effective vaccines and enhance CAR T-cells for the more precise and efficient targeting of tumor cells. However, CRISPR/Cas technology has challenges, including off-target effects, toxicity, immune responses, and inadequate tissue-specific delivery. Overcoming these challenges necessitates the development of a more effective and specific CRISPR/Cas delivery system. This entails strategically utilizing specific gRNAs in conjunction with robust CRISPR/Cas variants to mitigate off-target effects. This review seeks to delve into the intricacies of the CRISPR/Cas mechanism, explore progress in gene therapies, evaluate gene delivery systems, highlight limitations, outline necessary precautions, and scrutinize the ethical considerations associated with its application.

## 1. Introduction

The Clustered Regularly Interspaced Palindromic Repeats (CRISPR) and its associated protein (Cas) exists extensively in archaea and bacteria as an innate immune system. The rapid development of the CRISPR/Cas genome editing tools has revolutionized the gene therapy field and enhanced our ability to treat various genetically heritable diseases [1]. CRISPR was first identified by scientists while analyzing a gene for alkaline phosphatase in the prokaryotic *Escherichia coli* genome [2,3]. The sequence consisted of 29 nucleotide repeats broken up by 32 nucleotide spacer sequences that were only observed while the bacteria were in contact with exogenous DNA [3,4]. Prior to applying CRISPR systems to gene therapy, these unique repeats were exclusively used as a biomarker throughout the process of genotyping [5]. The Discovery of CRISPR across several bacterial and archaeal genomes led to the isolation of CRISPR-adjacent genes (Cas1-Cas4) that play a large role in our understanding of this mechanism’s editing ability [6,7]. The lactic acid bacteria *Streptococcus thermophilus* was later used to analyze the relationship between CRISPR and Cas proteins, revealing their coupled ability to provide prokaryotes with an advanced immune system for protection against bacteriophages and viruses [8].

## 2. Mechanism of CRISPR/Cas’s Action

**Figure 1:**
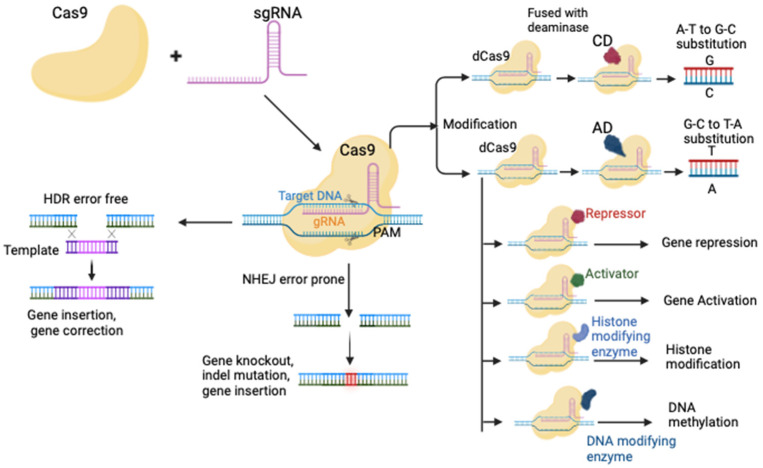
Mechanism of CRISPR action and gene function study. The CRISPR/Cas9 system creates a double-strand break in the target DNA, which can be repaired using NHEJ (non-homologous end joining) or HDR (homology-directed repair) pathways. NHEJ can result in frameshift mutations, insertion, or deletion of nucleotide bases, while HDR requires a homology donor template to correct mutations precisely and can insert or delete genes at specific locations in the genome. Additionally, Cas9 can be deactivated (dCas9) and fused with other proteins, such as a deaminase, transcription factor, or other proteins, to perform base editing, gene expression, imaging, or epigenome editing. An sgRNA that is about 130–150 nucleotides long guides the Cas effector to the precise genome cleavage site. The sgRNA is created by combining CrRNA and TracrRNA. The spacer within crRNA, which is only 18–21 nucleotides long, is the perfect complement to the target sequence. AD represents adenine deaminases, and CD represents cytidine deaminases. dCas, a modified Cas protein version, can be fused with AD or CD to achieve base editing. When dCas9 is fused with AD, it can convert G-C to T-A, while when fused with CD, A-T to G-C substitution occurs. With this capability, we can potentially correct harmful mutations and cure genetic diseases.

CRISPR sequences exist in various classes and species, containing different repeats and involved genes [9,10]. All types of CRISPR-Cas systems have been classified based on utilizing multi-subunit Cas-protein complexes (class I) or a single versatile Cas-protein (class II) [11]. The different types of CRISPR are described in Table 1. Studies have shown that class I CRISPR/Cas contains types I, II, and IV [12]. The system of interest in modern medicine and gene therapy is the class II CRISPR-Cas system, which includes type II, V, and VI [12]. The CRISPR-Cas9 and CRISPR-Cas12a effectors are type II and type V, respectively [13,14,15]. Type VI includes CRISPR-Cas 13 and is used for RNA editing [16]. Class 2, type V includes miniature CRISPR-Cas 12J, CRISPR-Cas 12f, and CRISPR-associated transposase [17,18,19]. Studies have shown that type I CRISPR-Cas are newly emerging tools for transcriptome and genome manipulation in eukaryotic cells [20,21] and microbiota [22]. The two essential components of the original Cas9 system from Streptococcus pyogenes (spCas9) include guide RNA (gRNA) and the CRISPR-associated Cas9 protein (1368 amino acids) [23]. The editing mechanism can be broken down into three phases: recognition, cleavage, and repair [24]. Recognition involves specialized gRNA capable of directing the Cas9 protein and detecting the genomic target sequence. The Cas9 nuclease acts as a scissor, creating a double-stranded break (DSB) at several nucleotides upstream of a complementary “protospacer-adjacent motif” (PAM) sequence [23]. Figure 1 illustrates the mechanism of CRISPR action and its application in different cellular processes. Cas9 is unable to cleave DNA in the absence of PAM. Structurally, the Cas9 nuclease consists of two active domains, RuvC and HNH (His-Asn-His), utilized for non-complementary and complementary DNA cleavage, respectively [25]. Cas9 contains a bilobed structure, an REC lobe (recognition lobe), and an NUC lobe (nuclease lobe). The REC lobe recognizes and binds to gRNA, and the NUC lobe consists of RuvC, HNH, and PAM interacting domains responsible for binding to target DNA [25]. The gRNA consists of CRISPR RNA (CrRNA) and trans-activating CRISPR RNA (TracrRNA). CrRNA contains a spacer sequence that recognizes target DNA by base pairing, while TracrRNA contains several loops to form a binding scaffold structure for Cas9 interaction. CrRNA and TracrRNA form single guide RNA (sgRNA) via an artificial linker [26,27]. SpCas9 recognizes the 5′-NGG-3′ sequence as a PAM in the target DNA [28,29]. Cas9 creates a DSB in target sequence [30] at a site 3 bp away from PAM. Following DSB, the DNA fragment of the bacteriophage or plasmid is expelled from the local genome, subsequently providing the host organism with immunity against invading mobile genetic elements. In the repair phase, non-homologous end joining (NHEJ) and homology-directed repair (HDR) pathways repair DSB lesions via host cell machinery [31]. DNA repair in the NHEJ process is mediated by inserting or deleting DNA base pairs. NHEJ is an error-prone repair pathway. An error can occur at any cell cycle phase, leading to insertion/deletion (InDel) mutations in target DNA [32,33]. In the presence of an appropriate sister chromatid donor, HDR allows for precise insertion or deletion, thus, error-free DNA repair (Figure 1). The HDR is most active in the late S2 and G2 phases of the cell cycle. The CRISPR/Cas system has recently been used for epigenome editing, prime editing, and base editing. Base editors are mainly classified into two classes: the adenine base editor (AD) and the cytosine base editor (CD) (Figure 1). The use of the CAS system in the cytosine base editor was reported in 2016 and has been used in many studies for base editing (A-T to G-C) in genomes [34,35,36,37]. Many studies have employed the CAS system as an adenine base editor (G-C to T-A) [38,39,40]. All types of transition mutations (G to A, T to C, C to A, and A to G) can be inserted into the genome using these AD and CD base editors [41]. The base editors can also be engineered to induce specific C to G transversion [42]. An engineered CAS, which is inactivated due to the insertion of mutations called dCAS [43], can be fused with a transcription factor to regulate gene expression [44,45], a repressor to repress the gene expression [43], a DNA modifying enzyme or histone modifying enzyme to perform epigenome editing [46,47], and genome imaging [48,49].

## 3. CRISPR and Other Genome Editing Methods

Since the discovery of CRISPR, several adaptations have been made to its molecular mechanism to enhance practices within biomedicine, agriculture, and others [5]. Through many clinical trials, CRISPR technology has been proven to have higher rates of efficacy for gene editing when compared to older gene therapy systems, such as zinc-finger nucleases (ZFNs) and transcription activator-like effector nucleases (TALENs) [4,53]. ZFN and TALEN tools rely on restriction enzymes and guide proteins, whose engineering is difficult, time consuming, and expensive. ZFNs are transcription factors that bind specifically to DNA sequences, and a new ZFN needs to be engineered for each new DNA targeted, which is a laborious and difficult process. TALENs are made from the fusion of a TALE (transcription activator-like effector) and the catalytic domain of restriction endonuclease FokI (Table 2), and their engineering is also difficult. ZFNs and TALENs use engineered proteins to target specific DNA sequences in the genome. In contrast, CRISPR/CAS uses a small segment of engineered guide RNA to drive the CAS effector to specific locations in the genome (Table 2). ZFNs and TALENs have an off-target effect on unwanted genes [54]. CRISPR-Cas is effective when provided with the correct template and relies on the development of specialized gRNA, which is easier to manufacture in comparison to the bulky protein guides required by ZFN and TALEN tools [55,56]. Table 2 compares and describes different features of CRISPR/Cas, ZFN, and TALEN systems. Additionally, under the right conditions, CRISPR-Cas can edit across plant, animal, and microbial genomes, posing advancements in genetic engineering across all living cells [56,57]. The CRISPR-Cas genome editing system was first used on animal cells in 2013 and poses biological risks and ethical concerns, as well as benefits [58]. CRISPR edits to the genome are irreversible and occasionally off-target. Mitigations to off-target effects rely on gRNA sequences that are complementary to possible off-target sequences. An additional issue with CRISPR-Cas editing falls on Cas nuclease binding and cleaving activity, as these processes are not consistently mutually exclusive. Mutations in the Cas9 protein can cause binding to occur without cleavage of target DNA [26]. Further research on the CRISPR-Cas delivery system is required to gain a full and complete understanding of its complications and potential side effects in order to expand on the role that CRISPR technology could play in genetic-based intervention therapies.

**Figure 2:**
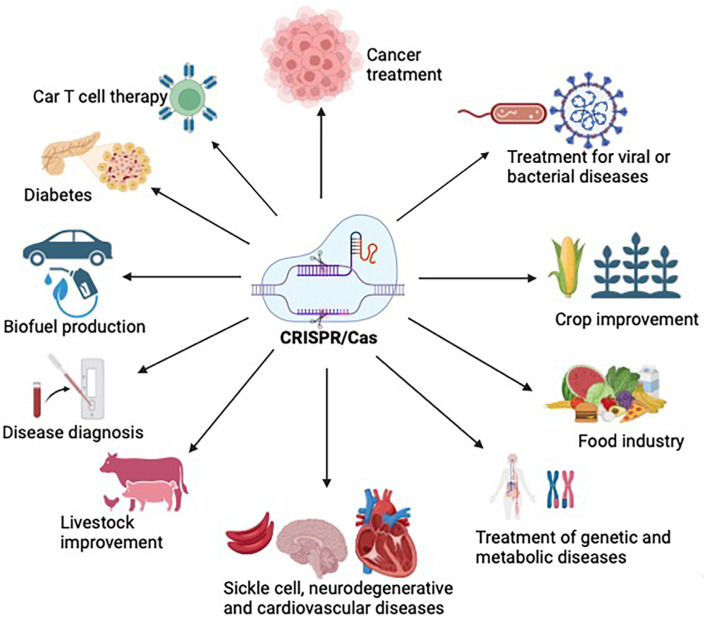
Versatile applications of gene editing with CRISPR/Cas. In the medical field, it has been applied to diagnose diseases and improve CAR T-cell therapy. In the food industry, CRISPR-Cas can enhance crop yields and improve resistance to pests. CRISPR technology has shown great potential in addressing genetic diseases through gene editing. In the field of plant biotechnology, CRISPR-Cas has been used to create crops that are more resistant to environmental stressors and have improved nutritional value. Furthermore, it has been applied to the production of biofuels and the improvement of livestock, resulting in more sustainable practices.

CRISPR genome editing technology has had a significant impact on various industries, including the biomedical, biotechnology, nanotechnology, plant, and livestock industries. One of the CRISPR applications is genome editing in the livestock industry to generate disease-resistant animals, improve animal welfare, and enhance productive traits [61]. Large animals, such as buffalo and cattle, have been the focus of this research. Moreover, CRISPR has proven to be useful in improving crops by enhancing their nutrient values [57,62]. CRISPR-based genome editing can potentially improve heart disease [63], neurological diseases [64], disease diagnosis [65], antiviral therapy [66], antibacterial disease [67], drug resistance [68], genetic disease [69], metabolic disease [70], biofuels [71], and many others. This technology holds great promise, and various industries are exploring its potential applications in different fields (Figure 2).

The CAS system has been used in agriculture to modify grain shape, size, and weight [72,73]. It has also been used to improve the size and shape of tomatoes and fruits for better consumption and nutrient values [73,74]. Moreover, engineering through the CAS system changed the color of fruits and flowers [75,76]. Furthermore, the CAS system increased the shelf life of crops, such as tomatoes and bananas [77,78,79]. The shelf life of bananas is affected by ethylene during the preservation process. Genetic engineering of MA-ACO-1 through the CAS system delayed fruit ripening by two days and increased the shelf life of bananas [80]. Humans cannot synthesize carotenoids in their bodies, and diet is the only source. Carotenoids are important for eye disease prevention and antioxidant processes.

Interestingly, CRISPR/CAS was used to insert CrtI and PSY genes into rice, increasing the β-carotene content to 7.9 μg/g in dry weight [81]. Another example of crop improvement through the CAS system is increasing the γ-aminobutyric acid (GABA) content in food. GABA is a neurotransmitter inhibitor and functions as a regulator of anxiety and blood pressure [82]. Researchers have deleted *SIGABA-Ts* and *SISSADH* genes through the CAS system, which increased GABA levels by ~20-fold in tomatoes [83]. There are many other examples of crop improvement through the CAS system, suggesting a wide application of the CAS system in agriculture (Figure 2). The CAS system has tremendous applications in livestock animals, improving the traits of large animals [84]. The CAS system has been used to produce disease-resistant animals [85,86], and it is also being used to control pests and insects [87,88].

## 4. Delivery Methods for CRISPR

**Figure 3:**
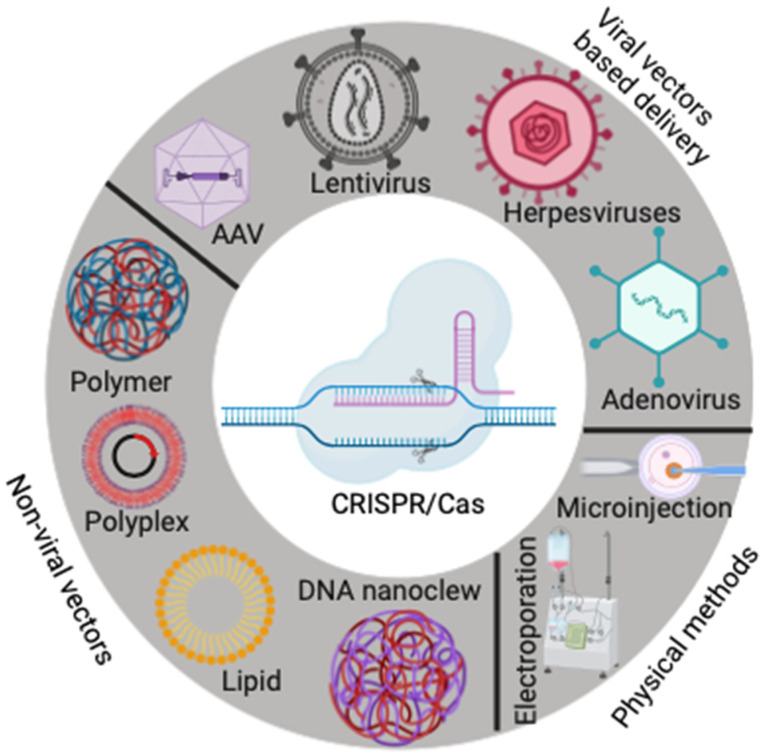
Various delivery methods for CRISPR/Cas. Some of the most promising viral vector-based delivery methods include AAV and LV. Physical methods, like microinjection and electroporation, have also shown great promise. In addition, non-viral vector methods, like nanoparticles, polyplexes, liposomes, and others, have been tested and have shown promising results.

The efficacy of CRISPR-Cas genome editing relies heavily on safe and predictable delivery systems in “in-vivo” (animals) [33,89]. Several delivery modes have been devised for integrating class II CRISPR-Cas-associated machinery into patients for therapeutic genome alteration (Figure 3). These delivery systems include physical, viral, and non-viral vectors, posing mechanism-specific benefits and potential side effects to CRISPR-Cas and recipient health [89,90]. Many of the viral vector delivery methods for CRISPR/Cas are pathogenic and invoke immune responses in the presence of the virus [89]. Heightened immune responses make subsequent deliveries difficult and less therapeutic to the patient [89,91]. Immunogenicity can be an additional drawback to viral-based delivery systems when a patient has previously been infected with a respective virus [91]. Non-viral delivery modes have been explored to combat immunogenicity and insertional mutagenesis issues accompanying viral-based delivery systems [89,92]. Non-viral delivery of CRISPR/Cas has been utilized in cancer treatments due to low pathogenicity and high biocompatibility with patient tissues [89,92]. The viral vs. non-viral delivery route depends on the clinical application (in vivo, in vitro), cargo size, and potential pre-existing patient immunity to viral vectors [89]. Despite potential side effects, viral vector delivery methods in gene therapy are favored due to a virus’ capability of crossing cellular boundaries and storing extracellular cargo [89,93].

Adeno-associated virus (AAV)-mediated CRISPR/Cas delivery has many benefits, including low pathogenicity, long-term gene expression, and both in vivo and in vitro applications [89,94]. AAVs have an icosahedral capsid, a 26 nm diameter, and a single-stranded genome with ~4.7 kb [95]. The AAV family is defined by its expansive serotype diversity and tropism, making it an attractive vehicle for gene therapy [89,96]. More than 3000 clinical trials that use AAV as a gene delivery vector are ongoing [97,98]. For example, a few gene therapies based on AAV vectors (Zolgensma, Luxturna, and Glybera) have been approved for human use [99]. AAV-mediated CRISPR delivery has been effective for both neural gene therapy and Duchenne muscular dystrophy (DMD) treatment in mice [100,101]. The AAV-mediated delivery of Cas9 and sgRNA to mouse models affected by a nonsense point mutation in the dystrophin gene (exon 23) successfully deleted the exon and subsequently decreased the DMD phenotype [102]. The CRISPR-associated protein SpCas9 is widely used for gene editing purposes. However, its size is a major limitation in packaging into a single AAV vector (packaging capacity is ~4.7 kb, including ITRs) [89,103]. As a result, the protein and sgRNA must be encoded on different vectors, which can pose a significant challenge for manufacturers [89,101,104]. However, alternative Cas proteins such as SaCas9, CjCas9, *Neisseria meningitides* Cas9 (NmCas9), *Streptococcus thermophilus* Cas9 (St1Cas9), and others are smaller in size and can be packaged into a single AAV, making gene therapy easier and more efficient [105,106,107]. Additionally, the discovery of compact Cas12f has opened many opportunities for scientists to use AAV as a delivery vector for various gene therapy and genome editing [108]. This is discussed in more detail in the miniature Cas section.

An alternative viral vector for the delivery of CRISPR-Cas is the retrovirus family, known as lentivirus (LV) (Figure 3), which has a single-stranded genome of 7–12 kb [109]. Similar to AAVs, LVs provide effective cellular transduction across various cell types (in vivo and in vitro), low immunogenicity, and safe delivery of gene editing machinery [89,110]. LVs for gene therapy were originally derived from the human immunodeficiency virus (HIV-1) and have since been altered for cell-specific transduction [111]. Despite being relatively easy to design, LVs typically integrate their genome into the host genome, subsequently elongating the process of gene expression [112]. The manipulation of LV via an integrase mutation has led to the development of “integration-deficient” lentiviral vectors, reducing the risk of continuous Cas overexpression and off-target effects [113]. Notably, the RNP (ribonucleoprotein) contains CRISPR-Cas and sgRNA and can be delivered by lentivirus or lentivirus-like particles without genome incorporation, thus eliminating the risk of off-target effects [114]. LV-associated delivery has been successful in preclinical trials for hemophilia gene therapy and Gaucher Disease [89,115,116]. The ex vivo LV-mediated delivery of CRISPR/Cas to hematopoietic and pluripotent stem cells has shown promise in phase I and II clinical trials [89]. The European Medicines Agency and the Food and Drug Administration (FDA) have approved LV for use [89]. This means that LV has met the safety and efficacy standards required by these regulatory bodies and is now authorized for medical treatments.

The non-viral vectors for the delivery of CRISPR-Cas have demonstrated several benefits, including minimal cost for wide-scale production, high biocompatibility, and low pathogenicity [117]. Lipid nanoparticles (LNPs) are amphiphilic molecules composed of ionized or cationic lipids engineered to deliver exogenous materials to cells, including nucleic acids and CRISPR-Cas machinery (Figure 3) [117]. Compared to other viral and non-viral delivery methods for gene therapy, LNPs pose advantages that make them attractive vehicles for administering cancer drugs, such as lowering drug toxicity and preventing drug degradation [118]. Cas and sgRNA mRNA, as well as the Cas:sgRNA RNP complex, have been correctly loaded onto LNPs and efficiently transported to the livers of mice for gene alteration [119]. The delivery of the CRISPR-Cas system via LNPs to tissues outside of the liver has yet to be efficiently explored and remains an issue when utilizing LNP delivery in preclinical settings [89]. To combat the COVID-19 pandemic, a highly successful mRNA vaccine was created to limit COVID-19 infection. Similar vaccine technology can be utilized to transmit CRISPR/Cas via LNPs, improving the efficacy of the solid nanoparticle delivery methods.

## 5. Miniature Cas12f

Neither SpCas9 (1368 amino acids) nor AsCas12a (1307 amino acids) can be packaged into a single AAV vector for efficient in vivo delivery. Although smaller Cas9, such as saCas9, cjCas9, nmCas9, etc., have been identified and used in all-in-one AAV vectors, packaging and transduction efficiency remain to be optimized [105,106,107]. Recently, researchers have identified super compact CRISPR/Cas effectors, such as Cas12f and Casϕ, which are classified as a type V-F system. These Cas effectors are 400–700 amino acids long and have shown genome editing activity in human cells [12,120,121]. The development of smaller CRISPR effectors and miniCAS shows great promise in overcoming the limitations of AAV and expanding the range of potential gene editing targets.

The first study identified Cas14 from an uncultured archaeon (Un1), initially named Cas14a1 and later renamed Un1Cas12f1, and evaluated the targeting specificity and activity using a single-stranded DNA in a PAM-independent manner but not in mammalian cells [17]. Several later studies engineered the protein and/or sgRNA scaffold of the Un1Cas12f1 system and demonstrated high editing efficiency on double-stranded DNA in a PAM-dependent manner and mammalian cells [18,122,123]. Un1Cas12f1 is also active for base editing [124] and gene regulation [125].

**Figure 4:**
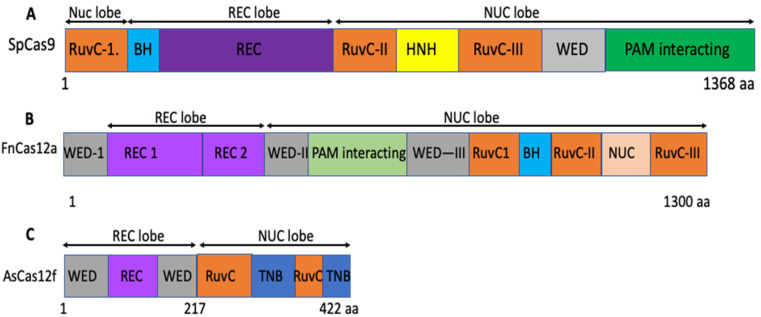
Size comparison of miniature CAS with other CRISPR effectors.

A recent study has identified a smaller Cas 12f version from *Acidibacillus sulfuroxidans* called AsCas12f (Figure 4). This version contains only 422 amino acids, has a PAM with TTR (where R is G or A), and has shown comparable genome editing efficiency in human cells [122,126]. This discovery is a game changer, opening exciting new opportunities for scientists to utilize the mini AsCas12f for genome editing in humans, with potential applications in gene therapies using AAV delivery vectors. Figure 4 illustrates the comparison of the length and size of three representative Cas enzymes—SpCas9, FnCas12a, and AsCas12f.

Recent studies on sgRNA engineering [122,124] and the structure of the Cas12f-sgRNA complex [127,128,129] have brought groundbreaking developments in genome editing research. Using cryoEM, researchers have uncovered the structure of AsCas12f, which forms a dimer structure similar to UnCas12f [108]. High-throughput screening using deep mutational sequencing (DMS) and structural design information has identified two AsCas12f variants, AsCas12f-HKRA and AsCas12f-YHAM, which have significantly enhanced genome editing activity of AsCas12f, called enAsCas12f, in mammalian cells, comparable to SpCas9 and AsCas12a, which is significant progress in the field [108]. Moreover, researchers have engineered an optimized sequence for sgRNA by deleting certain stem loops, which has led to better efficacy in human cells. To prove the application of enAsCas12f, the authors used an AAV vector to deliver enAsCas12f in human cells and determine the efficacy against six internal loci, such as VGFA, HEXA, PDCD1, TP53, and APOB, and found comparable editing to AsCas12a. EnAsCas12f also has comparable editing efficiency to SpCas9 and AsCas12a against the most promising therapeutic targets, such as PCSK9, ANGPTL3, and TTR. Furthermore, the researchers have demonstrated the efficacy of enAsCas12f in vivo using a mouse model.

The authors constructed a hepatotropic AAV serotype (AAV8) by inserting the AsCas12f or AsCas12f-HKRA variant under the HCRhAAT promoter and sgRNA (under U6 promoter) specific for the TTR gene (transthyretin amyloidosis). They then evaluated its therapeutic potential in 7-week-old mice by determining the expression of transthyretin in the plasma of the treated mice. Impressively, they found that the AsCas12-HKRA variant was able to dose–dependently reduce the level of transthyretin, with a high editing rate of approximately 66.3% achieved at the target location after only eight weeks of AAV-based gene delivery in the injected mice [108]. Next, the authors used the AsCas12f-HKRA variant to insert the EGFP gene at the Alb locus and found that there was a significant increase in the level of EGFP expression in the injected mice. They then applied a similar strategy to knockin coagulation factor IX at the Alb locus in hemophilia mice. They found that enAsCas12f increased the expression of factor IX in the plasma of treated mice [108]. Most significantly, they demonstrated high efficiency of factor IX positional knockin in mice using an all-in-one AAV vector encoding enAsCas12f and sgRNA and carrying a donor sequence (factor IX with both homology arms) with a self-cleaving sgRNA target site, which can eliminate the AAV vector after gene editing and knockin [108]. Finally, the scientists validated the efficacy of enAsCas12f in epigenome editing in mouse studies using an all-in-one AAV vector encoding denAsCas12f-VP64-PT2-MS2-P65-HSF1 and sgRNA in either universal CMV or liver-specific TTR promoters [108]. These groundbreaking developments in the Cas12f-sgRNA complex have the potential to revolutionize genome editing, paving the way for new possibilities in future research and therapeutic applications (Figure 4). The results of these studies are highly promising and will undoubtedly shape the future of genome editing research and particularly clinical trials.

## 6. Biomedical Applications of CRISPR/CAS

The CRISPR-CAS system has wide applications in biomedical sciences. The CAS system has been used to generate robust and reproducible model systems such as transgenic animals, brain organoids, IPS-derived cells, and cancer models to study various diseases, which was challenging before. For example, the CAS system produced a leukemia disease model by knocking out multiple genes simultaneously [130]. Indeed, the CAS system was used to generate iPSC-derived neurons that display characteristics and phenotypes of Alzheimer’s disease [131]. Creating an accurate disease model for Alzheimer’s disease is challenging. However, by leveraging the CAS system, it was possible to generate an accurate disease model for Alzheimer’s disease. Another biomedical application of the CAS system is high-throughput screening to better understand the cellular pathways and molecular mechanisms and identify genes involved in a disease [132,133]. The CAS system has been used to accurately diagnose diseases in a timely manner; this is very important for disease treatment and epidemiological monitoring of diseases. CRISPR-based diagnoses are accurate, fast, affordable, and can be performed at home. For example, CRISPR/CAS-based diagnosis methods, such as NASBACC (nucleic acid sequence-based amplification CRISPR cleavage), are ultrasensitive methods to detect Zika virus in the femtomolar range [134]. The CAS system was used to develop SHERLOCK diagnostic tools (specific high-sensitivity enzymatic reporter unlocking). SHERLOCK is currently being used to detect single DNA or RNA molecules in samples from dengue, Zika, and pneumonia patients [135,136]. SHERLOCKv2 is a highly sensitive method that detects target sequences at the femtomolar range (10^−21^). Moreover, the CAS system was utilized to develop DETECTOR (DNA endonuclease-targeted CRISPR trans reporter) diagnosis tools to discriminate between HPV16 and HPV 18 more precisely [137]. In biomedicine, the CAS system has been employed in treating hereditary diseases such as hemophilia [138,139], β-thalassemia [140,141], cystic fibrosis [142,143], Alzheimer’s disease [144], Huntington’s disease [145], Parkinson’s disease [146], Tyrosinemia [147], Duchenne muscular dystrophy [148], Tay–Sachs disease [149], Fragile X syndrome [150], phenylketonuria [150], blindness [151], cataracts [152], cancer [153], blood disorders [154], and others. The CRISPR system has been used to study and treat infectious diseases in humans [155]. There are many other biomedical applications of the CRISPR/CAS system. The CRISPR/CAS system has revolutionized the field of gene therapy. Figure 2 describes the various applications of the CRISPR/CAS system.

Recently, CRISPR-based technology developed STOPCovid.v2 to detect SARS-CoV-2 in infected patients. This detection method achieved a specificity of 98.5% and a sensitivity of 93.1%, which is higher than RT-PCR for SARS-CoV-2 detection. STOPCovid.v2 was approved by the FDA for SARS-CoV-2 detection [156,157].

## 7. CRISPR/Cas-Based Gene Therapies and Therapeutic Applications

The first CRISPR-based gene therapy in humans provided evidence for its application to treat both genetic diseases and infectious diseases [158]. About 6000 diseases are caused by genetic disorders, and the majority of them do not have effective treatment; thus, CRISPR provides an excellent opportunity to treat these genetic diseases. In 2020, Jennifer Doudna (University of California, USA) and Emanuelle Charpentier (Max Planck Unit for the Science of Pathogens, Germany) were awarded the Nobel Prize in chemistry for their work on endonuclease, the CRISPR-CAS system for gene editing. Gene therapy requires the replacement of a defective gene with a functional gene or the correction of mutations in a gene at its native location. A list of CRISPR-Cas systems that have been used in genome editing is described in Table 3. The Cas effector protein used, its classification based on the type and organism of origin, and the protospacer-adjacent motif (PAM) sequence that it recognizes are summarized in Table 3. In the SpCas9 system, sgRNA prefers the NGG PAM sequence, which is present in most organisms, thus facilitating genome editing in animals and plants [41,159]. The SpCas9 effector has been applied to many diseases, including cancer, sickle cell anemia, cardiovascular diseases, and neurodegenerative diseases [160,161,162,163]. Interestingly, SpCas9 was modified by mutating its structural domain to generate a dead Cas9 (dCas9) and fused it with other functional proteins such as an adenine base editor, a cytosine base editor, and prime editors to correct deleterious point mutations specifically. Similarly, other Cas effectors were identified and characterized as described in Table 3. Another example of CAS effectors includes Cas13a, which was isolated from Leptotrichia shahii and shown to have cleavage functions in many studies [41,164]. Compact Cas12f was also identified as having comparable genome editing to SpCas9 [108]. Notably, specific Cas9 variants have desirable properties that are utilized in various circumstances. For example, amongst the Cas9 proteins present in mammalian cells, XCas9 has a high PAM compatibility that helps with numerous processes within human cells [165]. Thus, the high-fidelity Cas9 variant is able to recognize stricter PAM sequences, which reduces the risk of unintended DNA cleavage from happening. Table 3 also mentions unique Cas effectors, such as Cas12a and Cas13. Cas12a is able to target single-stranded DNA specifically [122]. Cas13 targets RNA, which makes it valuable for cleaving RNA viruses, like HIV or influenza [166]. This diverse range of Cas effector proteins shown in Table 3 offers many advantages for CRISPR technology. Since different Cas effector proteins, such as Cas9, Cas12a, and Cas13, recognize and cleave distinct types of genetic material, researchers can choose from many options to select the most appropriate tool.

### 7.1. CRISPR Technology and Cancer

It is well known that each cancer patient has unique genetic and epigenetic variations that result in different reactions to the same treatment. CRISPR-Cas-based techniques have become a pivotal tool for advancing personalized treatment options in the context of cancer. CRISPR-Cas methods allow researchers to identify specific genetic factors influencing cancer development and formulate personalized therapeutics tailored to the individual [187]. CRISPR/Cas provided a powerful example of its therapeutic capacities by pinpointing and targeting the KRAS oncogene, subsequently preventing cancerous growth [188]. Before the advancement of CRISPR technology, gene editing tools, such as ZFNs and TALENs, were utilized, but they had many limitations [189].

Genome-wide CRISPR screening is a powerful tool for understanding and exploring the intricate contributions of diverse genetic elements to the complex landscape of cancer progression. The backbone of this method incorporated the usage of gRNA libraries, containing guide RNAs that are tailored for specific genetic elements [187]. CRISPR screens are deployed to navigate the genetics of different types of cancers by revealing regulators of genetic dependencies, synthetic lethal gene interactions, and drug targets across various malignancies [187]. Studies being executed on pulmonary cancer treatment state the prospects of CRISPR-CAS single handedly becoming the instrument for treating this disease by editing genes [189].

There are a few methods of the effective delivery of CRISPR-Cas gene editing for cancer therapy. Electroporation is a commonly used physical method that delivers CRISPR-Cas into tumor cells in vitro. Its main feature temporarily disrupts lipid bilayers, enhancing cell membrane permeability [188]. Viral vectors are another delivery mechanism but pose challenges for packaging large genes [188]. Non-viral vectors, such as commercial transfection reagents, have demonstrated promise in delivering CRISPR-Cas systems in vitro [188]. The ex-vivo delivery of CRISPR-Cas tools involves treating cells outside the body prior to implementation into the body [188]. In chimeric antigen receptor (CAR) T-cell therapy, a patient’s T-cells are genetically engineered to express receptors that recognize and bind to cancer cells. CAR T-cell therapy has shown success in treating various types of cancer, particularly B-cell malignancies [190,191]. The US FDA has approved CAR T-cell therapies for the treatment of B-cell lymphoma [192]. However, the use of this therapy on other types of cancer, such as solid tumors, is not as effective [193,194,195]. There are some challenges associated with the CAR T-cell approach, including cytokine-related toxicities, limited CAR T-cell expansion and persistence, and T-cell exhaustion. CAR T-cell hypofunction [196,197], limited CAR T-cell expansion and persistence, and premature senescence [196] are significant reasons for CAR T-cell therapy failure. CRISPR-Cas has been used to enhance T-cell persistence and effector function by removing negative regulators through gene editing [198,199,200]. The CRISPR-Cas system can also be used to precisely engineer CAR T-cells [201]. Studies have shown that targeting diacylglycerol kinase (DGK) using the CRISPR-Cas system can make CAR T-cells more resistant to immunosuppression [202]. Additionally, deleting genes responsible for cytokine release syndrome and neurotoxicity, such as IL-6 and GM-CSF, can improve CAR T-cell efficiency [203,204]. Interestingly, the CRISPR-Cas system has also been used to insert CAR cassettes at specific locations in the genome [205]. The diverse array of CRISPR-Cas delivery systems has underscored the ongoing exploration of innovative approaches to cancer gene therapy.

#### 7.1.1. Lung Cancer

Lung cancer is a complex disease that manifests in two primary forms: small-cell lung cancer and non-small-cell lung cancer [206]. Lung cancer has been identified as the second most diagnosed type of tumor, causing 1.8 million cancer-induced deaths [207]. Since cancer drug resistance has been the main culprit in providing relief to lung cancer patients, CRISPR techniques have offered a glimpse of hope for a better quality of life [206].

CRISPR-Cas systems are being utilized to edit oncogenic gene mutations, like EGFR [208]. EGFR is a tyrosine kinase, and mutations in its kinase domain are found in approximately 10% to 40% of non-small lung cancer patients. These mutations are mainly present in exon 19 and exon 21 of the EGFR gene [209,210]. A study has demonstrated that a CRISPR-Cas system and a donor template with homology arms can be used to replace mutated EGFR with normal or wild-type EGFR [211]. Moreover, the CRISPR-mediated knockout of mutated EGFR can prevent cancer cell growth and proliferation [206]. This gene elimination stops rapid cell growth and cell survival in vitro and in vivo [208]. CRISPR can also strategically insert genes that enhance cancer cell drug sensitivity [206]. Editing genes with cancer cells gives the opportunity to trigger apoptosis or even make the oncogenes vulnerable to alternative cancer therapies [208]. This approach effectively targets cancer cells and minimizes harm to healthy tissues. Researchers administered Adv-Cas9-sgG12S to A549 mice, resulting in a significant 30% reduction in tumor volume. The study also found that the expression of the mutant KRAS protein was extensively reduced, offering hope for those suffering from this devastating disease [212]. The use of a light-inducible CRISPR-Cas system to cleave the mutated BRAF gene is another significant development in cancer treatment. This approach induced melanoma cell apoptosis, leading to the inhibition of cell proliferation, migration, and invasion [213]. Patients with non-small lung cancer with CRISPR gene editing performed on their T-cells were observed. The off-target activity was only 0.05%, and edited T-cells showcased no adverse effects [208]. Compared to chemotherapy, CRISPR has been deemed more successful due to its ability to target epigenetic modifications in cancer and reverse the changes that make cancer cells proliferate and divide uncontrollably [214].

The possibilities of CRISPR have been marked beyond its therapeutic potential. With this, blood tests can utilize CRISPR to identify cancer cells’ gene signatures [214]. This personalized approach can help identify the disease at early stages when the most effective treatment can be administered [214]. The current perspective on CRISPR suggests that gene editing will be more efficient with continuous advancements in delivery methods that are employed in vivo [207]. CRISPR is gradually becoming a reliable tool for lung cancer due to its high efficacy in genome editing and minimal off-target activity [214].

#### 7.1.2. Brain Cancer

Glioblastoma multiforme (GBM) is a brain cancer that has been challenging to treat and has a poor prognosis. The emergence of CRISPR-Cas gene editing has led to recent advancements and promising brain cancer research and therapy. Some of the CRISPR-Cas-based clinical trials are listed in Table 4.

CRISPR methods are now making promising strides in silencing oncogenes. Researchers have utilized CRISPR technology to eliminate a specific gene in various cancer cell lines and monitor its impact on the growth of cancer cells. As a result, a DepMap database of cancer-related genes comprising data from thousands of cancer cell lines from numerous tumor types has been established [215]. CRISPR can potentially disrupt genes encoding growth factors, like EGFR and PDGFR [216]. By doing so, researchers can inhibit their pro-tumorigenic signaling pathways in an effort to starve the cancer cells of the growth signals they require to proliferate [216]. A study has employed a whole-genome CRISPR screen to identify the genes that are accountable for irradiation resistance in GBM cells. The authors of this study have used a human CRISPR activation library, which includes approximately 70,290 sgRNA that specifically targets 23,430 genes. The genes that are responsible for irradiation resistance in GBM cells were identified [217]. CRISPR is being used to edit genes encoding transcription factors that control gene expression. Specifically, MYC is an essential transcription factor that drives glioblastoma growth and survival [218]. CRISPR can target MYC and silence the gene in order to suppress further tumor progression [218]. CRISPR is being employed to insert genes encoding apoptotic proteins that are key for triggering cell death [219]. CRISPR can deliver the apoptotic gene p53 that eventually causes tumor cell death in glioblastoma models [219]. Utilizing this method directly eradicates cancer cells from the tumor mass. It is apparent that glioblastoma cells develop resistance to conventional therapies [220]. This occurrence is due to their robust DNA repair mechanisms [220]. CRISPR can overcome this complex design and disrupt genes involved in DNA repair [220]. CRISPR tools are used to edit immune cells with the help of chimeric antigen receptors [221]. These receptors are able to recognize and attack specific tumor antigens, which is essential for CAR T-cell therapy for glioblastoma treatment [221]. CRISPR offers a personalized approach for targeted therapies based on the patient’s tumor and unique genetic makeup [219]. The versatility of CRISPR gene editing makes it a promising method for halting the growth and proliferation of glioblastoma oncogenes [221].

### 7.2. Sickle Cell Anemia and Beta-Thalassemia

Sickle cell anemia is a genetic blood disorder that stems from abnormal hemoglobin, which is the culprit for health complications in the patient. Beta-thalassemia is another inherited blood disorder that disrupts beta globin production in the body. The emergence of CRISPR-based techniques has offered a new and advanced method to tackle these diseases.

Researchers have utilized CRISPR-Cas to pinpoint the BCL11A gene in stem cell patients with beta-thalassemia [222]. The gene editing in this clinical trial has allowed fetal hemoglobin production that makes reparations for the defective adult hemoglobin in the patient [222]. This system works by initiating a CRISPR screening to detect the regions of the HBG1 and HBG2 promoters [223]. These promoters are where the sgRNA sequences’ target sites are present, which serves as a guide for the Cas protein to locate and target the BCL11A binding sites [224]. CRISPR gene editing allows the repressor proteins (BCL11A and LRF) to switch on the production of fetal globin by preventing them from binding to the HBG1 and HBG2 promoters. This CRISPR technology has significantly improved red blood cell functions and patients’ overall well-being with transfusion-dependent B-thalassemia or sickle cell disease [222].

The challenges of using CRISPR include off-target editing and risks associated with off-target mutations [225]. CTX001, a form of CRISPR gene editing therapy, was observed to have high edited allele frequencies of 68.9% [222]. CRISPR techniques can pose issues with off-target mutations, severely disrupting normal gene functions [224]. Due to the long-term nature of CRISPR editing, the off-target activity could build up and showcase side effects in the patient’s body [224]. This issue can be overcome using rational sg RNA sequences and a more efficient and specific Cas protein [224]. Lack of knowledge surrounding the long-term side effects of CRISPR gene therapy necessitates careful and prolonged monitoring of patients. Due to these extensive hospitalizations, readmissions, and check-ups, many patients and family members have reported the financial strain that this therapy has caused them [225]. Additionally, it is crucial to note that gene editing success rates depend on the patient’s phenotype. Therefore, the results are different among each individual who receives CRISPR-based gene therapy [226].

### 7.3. Cystic Fibrosis

Cystic fibrosis (CF) is a genetic disorder that affects the lungs, pancreas, and other organs. The leading cause of CF is mutations in the Cystic Fibrosis Transmembrane Regulator (CFTR) gene. The emergence of CRISPR-Cas gene editing has alleviated some of the challenges endured by CF patients through therapeutic interventions [227].

CRISPR-Cas gene editing methods can be used to correct the mutated CFTR gene in the patient [227]. It is understood that this strategy can directly edit CFTR genes in the airway epithelial cells in order to restore the gene’s function [100]. CF patients also experience chronic inflammation and progressive lung damage. CRISPR techniques can be utilized to edit genes involved in inflammation pathways [228]. More specifically, this technology could complement existing iPSC-based approaches, which target lung epithelial cells relevant to CF lung disease [18]. Overall, this treatment helps to reduce lung damage and improves respiratory function in the CF patient.

Researchers are using the CRISPR-Cas technology and applying it to animal models, like knockout sheep, rats, and mice [227]. Animal models provide a platform for testing potential treatments and drugs before moving to human trials. CRISPR-generated CF models can be used to test gene editing therapies to correct specific mutations [227]. CRISPR’s versatility extends beyond directly targeting the CFTR gene. CRISPR can be used to edit genes involved in protein folding and trafficking [18]. This new avenue allows researchers to improve protein function, even when CFTR mutations are present [18]. CRISPR offers a promising approach for treating CF because it can be adapted to target a variety of CF mutations and personalize treatment based on the patient’s genetic makeup [18]. Prime editing makes this possible, where researchers are able to edit a specific DNA sequence of different lengths [229]. Since CFTR genes are large, prime editing can repair the disease-causing variants in an efficient manner [229].

### 7.4. Multiple-Sclerosis

Multiple Sclerosis (MS) is a chronic autoimmune disease that aggressively attacks an individual’s nervous system. This disease leads to progressive neurological deterioration. Emerging gene editing technologies, like CRISPR-Cas, offer relief for MS patients.

CRISPR methods are being used to edit genes in immune cells [230]. This gene editing inhibits healthy myelin from being attacked [230]. CRISPR techniques can be used to edit genes that are involved in myelin production and repair within oligodendrocytes [231]. Oligodendrocytes are the cells that are responsible for myelin formation [231]. This approach aims to stimulate the body to repair the damaged myelin naturally and halt the progression of the disease [231]. The candidate genes for CRISPR-Cas editing are IL7R, DDX39B, IL2RA, and TNFRSF1A [230]. Targeting these specific genes in immune cells makes it possible to reduce the overactive immune response that is a response to MS progression [230]. CRISPR serves as a major tool for research. Researchers are using CRISPR methods to create more precise models of MS by introducing mutations into the genomes of mice [232]. This approach allows researchers to study the models that closely resemble human MS and test potential therapies in a more relevant setting [232]. Autoreactive T-cells are the contributors to tissue injury in individuals with MS. Studying CRISPR methods now explains the immunological processes that dictate autoreactive T-cells [232]. Researchers can also use CRISPR methods to systematically disrupt genes in immune and nervous system cells [232]. This way, they can identify new genes that are involved in MS pathogenesis. An important feature of CRISPR is the ability to have personalized treatment strategies. CRISPR-based tools help identify genetic variations that contribute to a patient’s specific MS case [232].

Utilizing CRISPR methods requires careful evaluation and consideration of the potential risks and long-term repercussions [230]. Overall, MS development can be prevented and mitigated by correcting disease-associated mutations in specific genes.

### 7.5. HIV Gene Therapy

Human immunodeficiency virus, also known as HIV/AIDS, poses a significant health challenge to the human population globally. It is important to note that 38 million people are infected with HIV, and 800,000 people lose their lives due to HIV-related infections every year [233]. This virus attacks the body’s immune system and affects the quality of life of infected patients. No vaccine is available to cure HIV. The emergence of CRISPR-Cas gene editing techniques has allowed for better treatment options for people fighting against HIV.

CRISPR/Cas genome editing has been extensively employed to fight various types of viruses, including HIV [233,234,235,236,237]. Table 5 comprehensively summarizes various CRISPR-Cas-based gene therapies against HIV that target both viral and host factors. The studies have demonstrated significant progress in using the CRISPR-Cas system to remove proviral DNA from infected cells, which is a significant milestone in the fight against HIV [165,235,236,237,238]. The research has shown that focusing on highly conserved viral genes, such as the Rev gene, is a promising strategy, as it can eliminate the risk of virus escape and provide a more effective solution against HIV [239,240,241]. The stable transfection of cultured cells with plasmids containing spCas9 and duplex sgRNAs has shown great potential in excising proviral DNA from myeloid cells [128,235]. Moreover, the success of saCRISPR in eliminating HIV from infected cells by targeting multiple components of its proviral DNA, including 5′ LTR, 3′ LTR, and gag-pol, is a remarkable breakthrough in this field [238]. Lentiviral gene delivery of spCas9/sgRNA targeting the LTR U3 region to HIV-infected primary CD4^+^ T-cells from healthy human subjects and HIV patients [234] also induces effective HIV eradication. A combination of sgRNAs targeting the LTRs and the viral structural genes provides a more efficient means for HIV eradication in cultured cells [242]. The identification of a smaller saCas9 renders a rapid and extensive application of AAV in animal gene knockout [106,243,244] and knockin [245,246] studies. AAV9-mediated saCas9/duplex sgRNA can excise the integrated HIV genome in HIV-transgenic mice and rats [247]. AAV-DJ8-mediated quadruplex sgRNAs/saCas9 can excise HIV proviral DNA in several tissues and organs of HIV-infected hu-BLT mice [238]. Recently, the AAV9-saCas9/duplex sgRNA system was used to excise the SIV proviral genome in different organs/tissues, including the brain [248]. Studies have shown that RNA-targeted gene editing can eliminate latent HIV infection and prevent new virus production [235,242]. These studies clearly demonstrated the potential of CRISPR and its breakthrough application in disease treatment for infectious diseases. CRISPR-Cas can introduce fragmental deletion of proviral DNA in the infected cells [249]. This allows for the prevention of viral replication, which would help achieve a permanent cure for the virus [233]. CRISPR methods can also boost the immune system by editing the genome of immune cells, like T-cells [250]. Incorporating chimeric antigen receptors (CARs) allows CRISPR to distinguish and ultimately attack the infected cells [250]. This combination of CRISPR and Car T-cell therapy can be utilized for HIV treatment. Additionally, CRISPR can be used to edit genes involved in immune recognition pathways. This allows the immune system to be more efficient and likely to both detect and eliminate the HIV-infected cells in the patient’s body [251]. This approach aims to ultimately strengthen the patient’s natural defense mechanism against the virus [251]. When CRISPR-Cas systems are introduced into humans, there is a risk of subsequent immunogenicity in the patient [233]. To combat this, researchers can use a non-immunogenic Cas system, immune-suppressive drugs, or deliver the Cas proteins as mRNA [233]. Hence, combining different CRISPR-Cas systems to target the multifaceted HIV will be more effective if designed carefully. HIV may develop resistance against current drugs. CRISPR methods are employed to target the mutations that cause this level of resistance [252]. CRISPR is a personalized treatment method because it can strategically analyze individual patient genomes to identify specific viral mutations or the immune system’s weakness [250] and design personalized sgRNA for HIV proviral eradication. This way, patients can receive a specialized treatment option to fight against the virus present in their bodies. The development of 3D microglia-containing brain organoids to study HIV pathogenesis and CRISPR application in HIV eradication has been developed [253]. These 3D brain organoids represent valuable resources for studying HIV infection and latency and explore the effective delivery of the CRISPR/Cas editor for HIV eradication.

The CRISPR system has been used in many clinical trials for genome editing. CRISPR was used to disrupt the CCR5 gene in hematopoietic stem cells and progenitor cells obtained from patients to treat HIV-1 infection [268]. In another clinical trial, CRISPR-edited HSPC cells were engrafted into a 27-year-old man with HIV-1 infection. The results demonstrated that CCR5 gene disruption efficacy was increased from 5.2 to 8.3% in cells over 19 months (NCT03164135). Promising results were obtained from a recent clinical trial using CRISPR-based genome editing for sickle cell anemia [222]. In this study, CRISPR-based genome editing was performed in patient-derived HSPC cells targeting the BCL11A enhancer to produce an engineered HPSC called CTX001. Clinical trials were initiated to treat advanced and uncurable cancer, including multiple lymphoma and liposarcoma, by the CRISPR-based gene editing of patient-derived T-cells [269]. This study used CRISPR to suppress endogenous T-cell receptors (TCRs) and PD-1. Engineered T-cells were injected back into the patient, and three patients were treated using this method. A list of clinical trials that used CRISPR-based gene editing is described in Table 4.

### 7.6. Liver Diseases

The liver is essential for body homeostasis, drug detoxification, proteins, and lipid metabolism. CRISPR-based gene therapies have been used to treat many diseases that affect the function of the liver in the body. The most crucial liver disease is phenylketonuria (PKU), caused by a mutation in PAH, which encodes for phenylalanine hydrolase (PAH). PKU is an autosomal recessive liver disorder in which the level of phenylalanine increases in the blood when PAH activity is reduced, leading to neurotoxic effects. In severe cases of PKU, a point mutation Arg408Trp in PAH is the most prevalent [270]. When PAH activity is reduced, the level of phenylalanine in the blood increases, leading to neurotoxic effects. A recent study used an rAAV vector and CRISPR with a cytidine base editor to correct PAH mutation. About 63% of the mRNA correction rate was achieved, and a normal phenylalanine level was restored post-gene therapy in these mice [271]. Treated mice showed reduced growth retardation after genome editing in the homozygous Pah^enu2^ mouse model. Wilson’s disease (WD) is a rare genetic disorder caused by a mutation in the ATP7B gene, a copper-transporting P-type ATPase. AAV-based gene therapy in the WD mouse model reduces the copper level in these mice after six months of administration of gene therapy [272]. In another study, hepatitis B virus (HBV)-infected cells were targeted by the CRISPR/CAS9 system using nanoparticles to inactive HBV covalently closed circular DNA [273]. CRISPR/CAS-based gene therapy has been employed to treat other liver diseases such as Tyrosinemia type I (HT1) [245], Argininemia [274], Alpha-1 antitrypsin deficiency (AATD) [275], hemophilia [276], and ornithine transcarbamylase (OTC) deficiency [104,277].

### 7.7. Cardiovascular Diseases

Cardiovascular diseases are responsible for death in humans globally [278]. Cardiovascular diseases that affect humans include heart attack, aortic dissection, atherosclerosis, and myocardial hypertrophy [279,280,281]. Direct CRISPR delivery to cardiomyocytes may lead to off-target effects. Using CRISPR technology, a dCAS9/VPR transgenic mouse was developed, where dCAS9/VPR was expressed using cardiomyocyte-specific promoter Myh6 [282]. The administration of AAV9 harboring sgRNA activates gene transcription that promotes cardiomyocyte proliferation. This study holds great promise because cardiomyocyte proliferation decreases with aging, which can be restored using CRISPR-based gene therapies. In another study by Zhang et al., CRISPR was specifically delivered to vascular smooth muscle cells using a hydroxyl-rich lipid nanoparticle [283]. A study has specifically delivered CRISPR to endothelial and VSMCs using lipid and polymer nanoparticles [284].

## 8. CRISPR and Drug Approaches

Medicine is experiencing a transformative shift as CRISPR-Cas gene editing technologies gain popularity. This powerful tool is being used alongside traditional drug development approaches. Combining these methods allows researchers to discover avenues for tackling several complex diseases.

It is vital to point out that CRISPR techniques enhance drug sensitivity. CRISPR edits genes involved in drug resistance pathways, which makes cancer cells more prone to traditional therapies [285]. A considerable example is the ability of CRISPR to enhance the effectiveness of chemotherapy drugs that are involved with leukemia treatment [285]. With this, patients’ bodies can overcome resistance, and the treatment’s outcomes are greatly improved. CRISPR methods are considered to be personalized medicine. This is because CRISPR genome-wide screening identifies drug targets by disrupting genes and observing their effects on disease progression [232]. This means that CRISPR allows for discovering new therapeutic advancements geared towards specific disease mechanisms [232]. Additionally, CRISPR has the ability to engineer cells to behave like drug delivery vehicles [286]. This approach is valuable because CRISPR-edited macrophages can now deliver drugs directly to tumors [286]. This poses a safe and efficient technique due to minimal side effects on healthy tissues [286]. Researchers can create libraries that have many varying CRISPR gRNAs that target genes [287]. These libraries are important because they can be utilized to screen collections of potential drugs [287]. This CRISPR approach greatly speeds up the drug discovery process. CRISPR-mediated gene editing is also known for its precision when editing genes related to drug discovery in cells or animal models [287]. Therefore, CRISPR approaches validate drug targets by ensuring the drugs only affect their relevant pathways [287].

## 9. CRISPR/Cas and Vaccine Development

**Figure 5:**
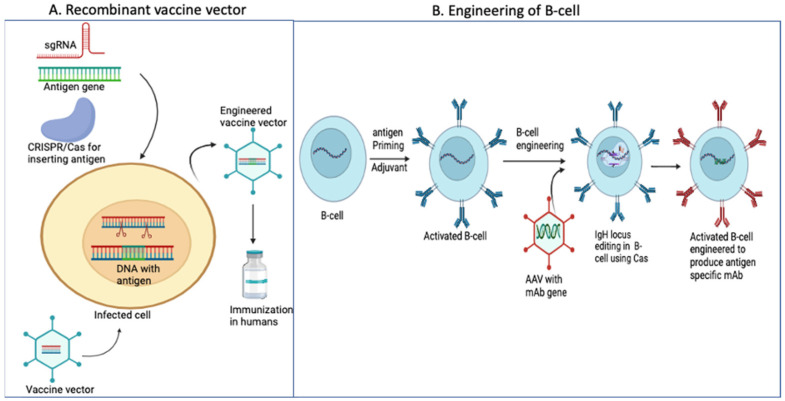
The role of the CRISPR/Cas system in developing vaccine vectors and engineering B-cells to produce new-generation vaccines. (**A**) CRISPR/Cas can precisely manipulate the genetic material of viruses to create safe and effective vaccines. (**B**) The CRISPR/Cas system has also been used to engineer B-cells, which are immune cells that produce antibodies. By modifying B-cells using the CRISPR/Cas system, researchers can create new-generation vaccines that are more effective and targeted than traditional vaccines.

The CRISPR/Cas system is a revolutionary technology that has been used to generate recombinant vaccines with greater efficacy, lower cost, and less effort than conventional methods. These traditional techniques, such as gene cloning, bacterial artificial chromosomes (BAC), cosmids, recombinant plasmids, and viral vector-based vaccines, are time consuming, costly, and labor intensive and may have lower efficiency [288]. In contrast, the CRISPR/Cas system can produce multivalent vaccines that offer long-lasting and protective immunity and have tremendous potential in generating specific B-cells to produce highly potent antibodies against current and future emerging strains of viruses (Figure 5A). The CRISPR/Cas system has been used to produce recombinant vaccines for various organisms, such as viruses [289], bacteria [290], fungi [291], plants [292], and animal cells [293]. Cas systems can be used to knockout or insert new genes in the cells or viral vector to develop a new generation of vaccines. Indeed, the CRISPR system has been used to edit virus genomes to develop recombinant virus vaccines [288,294]. For example, the Herpesvirus of Turkey (HVT) is a widely used virus vector used for developing vaccines against various diseases, including Marek disease, a common herpes infection in turkeys [295]. However, the live HVT vector was found to have low immunogenicity, implying that it was not generating a strong immune response. To overcome this issue, researchers have taken advantage of CRISPR/Cas technology to introduce genetic mutations in three genes, gE, gB, and gI, of the HVT vector that displayed a significantly higher level of immunogenicity, indicating a greater potential to stimulate the immune system to fight off infections [295]. CAS technology was utilized to create a recombinant HVT vaccine vector for infectious bursal disease (IBD) by inserting a VP2 cassette at the UL54-UL56 locus [295]. The results of this study showed that Cas can effectively generate recombinant vaccine vectors without causing off-target effects [295]. CRISPR/Cas technology was used in an elegant study to create a multivalent recombinant HVT vector that expresses several antigens. The researchers used CRISPR/Cas to insert gIgD and H8N2 in the HVT-VP2 vector, resulting in a recombinant multivalent vaccine that can target different avian pathogens [296]. A recent study has utilized the CRISPR/Cas system to create a bivalent recombinant duck enteritis virus (DEV) by inserting hemagglutinin (HA) protein into the UL26 locus of DEV. This study has proven this method to be promising [297]. Another study has also utilized the CAS system to create trivalent recombinant DEV by inserting premembrane protein (PrM) and glycoprotein E (gE) for duck tembusu virus (DTV) and HA for avian influenza. This was performed to prevent both avian influenza and DTV [298]. Figure 5A depicts a strategy that can be used to create recombinant virus vaccine vectors. The use of CRISPR for inserting foreign genes into viral vectors has revolutionized the field by offering an easy, efficient, and time-saving method. By employing CRISPR, researchers successfully inserted the green fluorescence protein into the pseudorabies virus (PRV) genome, enabling the visualization of the virus using microscopy. This breakthrough not only enhances the effectiveness of antiviral drug screening but also streamlines vaccine production [299]. Furthermore, the CRISPR system facilitated the insertion of a DNA sequence of over 4 kb into the PRV genome for vaccine development [300]. Using the CRISPR system, a vaccine strain of the PRV was developed by deleting TK (thymidine kinase) and gE genes [301]. A triple gene deletion of TK/gI and gE in the PRV led to the development of a fully attenuated vaccine that provided protection against infectious PRV strains [302]. Moreover, the CRISPR/Cas system was used to generate a multivalent recombinant vaccine by deleting virulence factors from the virus genome and inserting a heterologous gene. For instance, CRISPR was employed to knockout the US4 (unique short 4) and TK genes from the infectious laryngotracheitis virus (ILTV) genome and then insert the F (fusion) gene from the Newcastle disease virus (NDV) to form the multivalent recombinant vaccine [303]. Additionally, in a separate study, CRISPR was utilized to create a recombinant canine distemper virus (CDV) by expressing the F, H, and M (matrix) genes in CDV virus-like particles. The results demonstrated that the recombinant CDV-VLP triggered a robust immune response compared to the parental virus in minks and foxes [304].

The CAS system has been used to create a recombinant vaccinia virus, which is a popular viral vaccine vector, to produce MAVERICK. MAVERICK is a marker-free vaccinia virus that has been engineered using the CAS system and has been used to create vaccines [305,306]. Moreover, the CAS system has recently been utilized to produce recombinant phages by inserting SARS-CoV2 envelope, spike, and nucleocapsid genes in T4 phage. This process has led to the development of a recombined T4 phage that has shown promising results in inducing a robust T-cell immune response and providing complete protection against virus infection [307,308]. CRISPR/Cas has been utilized in recent research for various applications, including editing the DNA virus genome for vaccine development. The study demonstrated an increase in genome editing through HDR mediation using the NHEJ inhibitor SCR7 in the CRISPR/Cas9 system [309]. It has been shown that two copies of ICP0 at different locations in the HSV-1 genome could be sequentially edited by the CRISPR/Cas system. These targeted DNA virus editing applications suggest the potential use of the Cas system in developing recombinant virus vaccines [309]. Furthermore, researchers have used the CRISPR/Cas system to activate latent HIV by targeting the long terminal repeat activator site using dCas9 and the transcriptional activator VP64 [310]. These studies are valuable for the potential sterile cure of virus infections, as the CRISPR-mediated activation of latent HIV-1 can make patients more receptive to antiviral therapies. Moreover, CRISPR/Cas has been employed to edit parasite DNA to create genetically modified live attenuated whole parasite vaccines [311]. For instance, it was used to produce a second-generation Leishmania vaccine by creating genetically attenuated Leishmania major, which reduces infectivity and can activate a robust long-term immune response without causing illness [311]. Leishmania is transmitted by the bite of an infected sand fly.

In response to the emerging SARS-CoV-2 and COVID-19 pandemics, researchers have turned their attention to using the CRISPR/CAS system to target RNA viruses. The Cas system has been used to inhibit the replication of RNA viruses in infected cells [312]. Cas13 enzymes are part of the class II type VI CRISPR system and have RNA-targeting activities to cleave RNA in human and plant genomes. These CAS nucleases can target both single-strand RNAs (ssRNA) and double-strand RNA viruses [312]. Notably, studies have reported that the Cas9 system from Francisella can be used to edit ssRNA [313]. CAS13d has been shown to inhibit SARS-CoV-2 and influenza virus replication in human lung epithelial cells. In this study, the authors identified six crRNAs that can target about 90% of all coronaviruses, marking a significant breakthrough in the field [311].

B-cells are an essential component in the development of vaccines. They produce immunoglobulins and cytokines that are critical to building immunity and remain present in the body throughout one’s life as plasma cells. The CRISPR/Cas system has wide-ranging applications in engineering B-cells (Figure 5B) [314,315]. In fact, studies have shown that the Cas engineering of B-cells can increase their numbers by an incredible 10 folds, with about 80% of PBMC being B-cells [314]. Primary B-cells can be electroporated between days 3 and 7 post-stimulation, making them highly compatible with this technology [314]. The CRISPR/Cas technique has been used to introduce frameshift mutations in the papain Fc cleavage site, resulting in Fab secretion instead of complete IgG [316]. In murine hybridoma, a CRISPR-mediated class switch recombination of 4–11% has been achieved [316]. Furthermore, CRISPR-mediated gene editing can identify important factors responsible for V(D)J recombination, which is a crucial mechanism for developing immune responses [317]. Genome editing in activated B-cells has been found to be more efficient due to the upregulation of DNA repair in rapidly proliferating cells [318]. Scientists have used the CRISPR/Cas system to modify B-cells at the IgH and IgK locus to produce HIV-specific broad-neutralizing antibodies. They found that these engineered B-cells are better in terms of clonal expansion, class switching, and memory retention [319,320,321]. In another study, the native heavy chain region of the B-cells was replaced with the PG9 antibody, an anti-HIV antibody. The results showed that human primary B-cells engineered in this way could produce antibodies that bind with gp120 and can undergo affinity maturation [322]. These studies show that CRISPR/Cas technology can modify B-cells’ genetic material to create highly effective vaccines that target specific diseases. By precisely targeting and modifying B-cells, it is possible to enhance their ability to recognize and fight off pathogens, leading to the development of vaccines with improved efficacy (Figure 5B).

## 10. Conclusions and Future Direction

CRISPR/Cas has been widely used and implemented in many applications for precise genome editing in humans, animals, microbes, plants, and others. It is adapted from the innate immune system of microbials. This technology is highly effective and precise, with minimal off-target effects, making it an excellent tool for correcting genetic mutations. The CRISPR/Cas system has shown great promise in treating genetic diseases, such as sickle cell disease (SCD), thalassemia (TDT), X-linked diseases, eye diseases (ocular gene therapy), and others. CRISPR/Cas has also significantly contributed to cancer research. By knocking out oncogenes and activating tumor suppressor genes, CRISPR/Cas has helped researchers discover novel drug targets for cancer treatments. Moreover, it has been used to delete the genome of oncogenic viruses, such as HPV, which can reduce the incidence of tumor formation in vulnerable populations. Studies have shown that CAR T-cell therapy can effectively improve by inhibiting related genes using CRISPR/Cas editing. CRISPR/Cas can be used to modulate the genome, epigenome, or transcriptome of tumor cells, and it can also be used to produce effective cancer gene therapy. CRISPR/Cas can be delivered using viral and non-viral vectors, with large-capacity viral vectors, such as cytomegalovirus-based vectors, serving as an all-in-one vector delivery for large-sized CRISPR and sgRNA [323,324,325]. The CRISPR/Cas system can also be used to cure HIV. Studies have shown that the HIV genome can be removed using CRISPR/Cas for sterilizing the cure of HIV, which will benefit millions of people and prevent disease transmission. Due to high specificity and efficacy, the CRISPR/Cas system has been utilized to generate recombinant vaccines and B-cell engineering to produce more effective vaccines [289,326]. CRISPR/Cas system-based genetic engineering can be used to increase the vaccine yield by removing negative factors that suppress the host immune system. Notably, CRISPR/Cas-engineered vaccines are more effective and require a lower cost and time to generate than conventional vaccines [288,289].

Off-target effects or unintended cutting by CRISPR/Cas is still a major concern when using CRISPR-based gene therapy in clinical and therapeutic applications [327,328]. This unintended cutting can cause harmful mutations or genetic changes that have serious consequences. However, designing highly specific gRNA for target genes and improving the Cas/sgRNA complex can help eliminate the off-target effect of CRISPR. Using the CRISPR-Cas RNP direct delivery to target cells will greatly reduce off-target effects since the RNP degrades after gene editing [329]. In addition, increasing the delivery efficiency and selecting all-in-one vector delivery methods can also reduce off-target effects and production costs. Currently, most clinical trials use CRISPR/Cas in ex vivo patient-derived cells. These cells are then engineered and injected back into patients, which reduces the potential off-target effects of CRISPR. As most clinical trials for CRISPR-based gene therapy are ongoing, we expect to have a comprehensive understanding of the efficiency and specificity of this technology in patients. One of the major challenges for CRISPR-based gene therapy is delivering it in vivo to specific organs or cells in patients. Due to the lack of robust and effective in vivo delivery tools, this therapy is currently limited to only a few diseases. Another significant challenge is the cost, as approved gene therapies are expensive and are not affordable for most people worldwide. For example, CAR T-cell immunotherapies, like Tisagenlecleucel and Axicabtagene ciloleucel, approved by the US Food and Drug Administration (USFDA), can cost around USD 475,000 and USD 373,000, respectively. In vivo CAR-T or CAR-NK therapy with CRISPR/Cas technology is a promising solution. Another gene therapy named Luxturna^®^, used to treat eye diseases caused by mutations in the RPE65 gene, costs approximately EUR 410,550. These gene therapies are not affordable for most people, particularly in Asia, the Middle East, and African countries, where vulnerable populations are predominantly affected. However, CRISPR/Cas may not be effective in treating diseases with multiple mutations, such as cancer, compared to monogenic diseases. Although CRISPR/Cas can correct multiple mutations, off-target effects could lead to the deletion of large DNA fragments or unwanted gene editing. In summary, the potential of CRISPR-based treatments is vast and can play a significant role in biomedicine, with applications in different areas of medicine and biotechnology.

## Figures and Tables

**Table 1:** Different types of CRISPR and their properties [12,50,51,52].

CRISPR Types	Complex Effector	Target	Protein	Properties
Type I	Class 1 (multi-subunit)	DNA	Cas3	ssDNA cleavage
Type II	Class 2 (single subunit)	DNA	Cas9	Blunt DSB
Type III	Class 1 (multi-subunit)	DNA/RNA	Cas10	RNA molecule binding
Type IV	Class 1 (multi-subunit)		Csf1	
Type V	Class 2 (single subunit	DNA	Cas12	Staggered DSB
Type VI	Class 2 (single subunit)	RNA	Cas13	RNA guided RNase

**Table 2:** Functional evaluation of CRISPR, ZFNs, and TALENs [4,53,54,59,60].

	CRISPR/Cas9	ZFN	TALEN
Enzyme	Cas9 and different variants	FokI	FokI
Target recognition	RNA-DNA interaction, sgRNA	Protein–DNA interactions, RVD repeats	Protein–DNA interaction
Delivery	Cas9 protein with sgRNA complementary to the target sequence	Two ZFNs for the target sequence	Two TALENs for the target sequence
Specificity of target DNA	18–21 bp and NGG (PAM)	(9 or 12 bp) × 2	(8–31 bp) × 2
Benefits	Affordable and rapid	Time consuming and resource intensive	Time consuming and affordable
Preparation	sgRNA synthesis or cloning	3–4 zinc-finger domains	8–31 repeats
Construction	20-nucleotide sgRNA sequence construction specific to each target	Require engineering of protein for every single target	Require protein engineering for every single target

**Table 3:** Some of the Cas effectors used in genome editing.

Name	Cas Protein	Type	Organism/Resource	PAM Location	PAM	Reference
FnCas9	Cas9	Type II	*Francisella novicida*	3′	NGG	[167]
CjCas9	Cas9	Type II	*Campylobactor jejuni*	3′	NNNNRYAC	[168]
AsCas12a	Cas12a (cpf1)	Type II	*Acidaminococcus* sp.	5′	TTTV	[169]
FnCas12a	Cas12a (cpf1)	Type II	*Francisella novicida*	5′	TTTN or YTN	[169,170]
FnCas9 Variant	Cas9	Type II	Modified FnCas9	3′	YG	[167]
evoCas9	Cas9	Type II	Mutated SpCas9	3′	NGG	[171]
NmCas9	Cas9	Type II	*Neisseria meningitidis*	3′	NNNNGATT	[172]
LsCas13#	Cas13 (C2c2)	Type VI	*Leptotrichia shahii*			[166]
SpCas9	Cas9	Type II	*Streptococcus pyogenes*	3′	NGG	[26,27]
St1Cas9	Cas9	Type II	*Streptococcus thermophilus*	3′	NNAGAAW	[173,174]
LbCas12a	Cas12a(cpf1)	Type II	*Lachnospiraceae bacterium*	5′	TTTV	[169]
SaCas9	Cas9	Type II	*Streptococcus aureus*	3′	NNGRRT	[106]
St1Cas9	Cas9	Type II	*Streptococcus thermophilus*	3	NGGNG	[173,174]
Cas14	Cas14		*Archaea*			[17]
eSpCas9	Cas9	Type II	Engineered SpCas9	3′	NGG	[175]
Modified SpCas9	Cas9	Type II	Engineered SpCas9	3′	NAG or NGA	[176]
SpCas9 HF	Cas9	Type II	Engineered SpCas9	3′	NGG	[177]
SpCas9 NG	Cas9	Type II	Engineered SpCas9	3′	NG	[178]
SaCas9 KKH	Cas9	Type II	Engineered SpCas9	3′	NNNRRT	[179]
xCas9	Cas9	Type II	Engineered SpCas9	3′	NG	[165]
HpaCas9	Cas9	Type II	Mutated SpCas9 HF	3′	NGG	[180]
Sniper Cas9	Cas9	Type II	Engineered SpCas9	3′	NGG	[181]
SpRY	Cas9	Type II	Engineered SpCas9	3′	NYN or NRN	[182]
Cas9-NRNH	Cas9	Type II	Engineered SpCas9	3′	NRNH	[183]
SpG	Cas9	Type II	Engineered SpCas9	3′	NGN	[182]
AsCas12f1	Cas12f	Type V-F	*A.sulfuroxidans*	5′	NTTR	[122]
enOsCas12f	Cas12f	Type V-F	*Oscillibacter* Sp.	5′	NTTN	[184]
enRhCas12f	Cas12f	Type V-F	*Oscillibacter* Sp.	5′	CCN	[184]
CnCas12f	Cas12f	Type V-F	*Clostridium novyi.*	5′	CCN	[185]
enAsCas12f Variant	Cas12f	Type V-F	*Acidicacillus supfuroxidans*	5′	NTTR	[108,129]
AcCas12n	Cas12n	Type V-U4	*Actinomadura craniellae*	5′	NAAN	[186]

**Table 4:** CRISPR-based gene editing in clinical trials.

Disease or Conditions	Status as of March 2024	Intervention	Reference
Hematological malignancy	Active	Non-interventional	NCT06208878
Gastrointestinal cancer	Active	Drug	NCT04426669
HIV infection	Active	CCR5 gene modification EBT101	NCT03164135, NCT05144386
Pulmonary tuberculosis detection	Unknown	CRISPR-based test	NCT04074369
Sickle cell disease	Completed		NCT03167450
Viral keratitis	Completed	Drug BD111	NCT04560790
Age-related macular degeneration (AMD)	Active	Genetic HG202	NCT06031727
Leukemia	Active	Genetic: XYF19 CAR T-cell	NCT04037566
Multiple myeloma	Active	Biological: CTC120	NCT04244656
Beta-thalassemia	Active	Biological: CTX001	NCT03655678
Renal cell carcinoma	Active	Biological: CTX130	NCT04438083
Beta-thalassemia	Active	Biological: VGB-Ex01	NCT06041620
Cervical carcinoma	Active	Biological: CTX131	NCT05795595
Non-Hodgkin’s lymphoma	Active	Biological: CTC112	NCT05643742
Kabuki syndrome 1	Completed	Genetic: intervention on primary cultured cells	NCT03855631
Esophageal cancer	Completed	Other: PD-1 knockout T-cells	NCT0308171
Sickle cell disease	Active	Genetic: nula-cel drug product	NCT04819841
Non-Hodgkin’s lymphoma	Active	Biological: TRAC and Power3 genes knockout allogeneic CD19 targeting CAR T-cell	NCT06014073
COVID-19 test	Completed	Diagnostic test: proof lab test	NCT05331976

**Table 5:** CRISPR-Cas-based gene therapies for HIV.

CRISPR-Cas System	Gene Target	Delivery Methods	Result	References
Cas9	A3B, A3G (host)	LV	Restriction factors in the host were activated	[254]
Cas9	CCR5 (host)	AAV	Viral replication was inhibited	[255,256,257]
Cas9	B-cells (Host)	AAV	Inducible expression of anti-HIV broad neutralizing antibody	[258]
Cas9	CXCR4 (host)	LV	Viral replication was inhibited	[259]
Cas9	Tethrin promoter (host)	LV	Inhibition of viral replication	[260]
Cas9	Env (virus)	LV	HIV elimination	[261]
Cas9, Cas13d, Cas13a, Cas12a	Gag (virus)	AAv, LV	HIV eradication, latent proviral DNA inactivation	[236,237,238,241,242,261,262]
Cas13a, Cas9	Rev (virus)	LV	Latent proviral DNA inactivation and HIV eradication	[239,240,241,261]
Cas12a, Cas9	LTR (virus)	LV	Virus multiplication inhibition	[235,237,263]
Cas13a, Cas13d, Cas9	Pol (virus)	AAV, LV	Virus production inhibition	[241,242,262,264]
Cas12a, Cas9	Tat (virus)	LV	Virus elimination	[237,239,261,265,266]
Cas12a	Nef (virus)	LV	Inhibition of virus production	[267]

## Abbreviations

Ad: adenovirus, AAV: adeno-associated virus, CRISPR: Clustered Regularly Interspaced Short Palindromic Repeats, crRNA: CRISPR RNA, dCas: deactivated Cas, FDA: Food and Drug Administration, HIV: human immunodeficiency virus, LV: lentivirus, LNP: lipid nanoparticle, sgRNA: single guide RNA, DSB: double-stranded break, HDR: homology-directed repair, NHEJ: non-homologous end joining, PAM: protospacer-adjacent motif, TracrRNA: trans-activating CRISPR RNA, ZFN: zinc-finger nuclease.
